# Causal relationships between obesity and pancreatobiliary diseases: a two-sample Mendelian randomization study

**DOI:** 10.1007/s40519-023-01592-x

**Published:** 2023-08-01

**Authors:** Dan Huang, Yu Liu, Wenjun Gong, Jian Zou

**Affiliations:** Department of Clinical Pharmacy, The People’s Hospital of Pengzhou, No. 255 Second section of South Third Ring Road, Chengdu, China

**Keywords:** Body mass index, Waist circumference, Hip circumference, Waist-to-hip ratio, Obesity, Pancreatobiliary diseases, Mendelian randomization

## Abstract

Previous observational studies have investigated the relationship between obesity and the biliary tract and pancreas. The causality, however, is still to be confirmed. This study was designed to explore the causality between obesity which included body mass index(BMI), circumference (WC), hip circumference (HC) and waist-to-hip ratio (WHR), and pancreatobiliary diseases with a Two-Sample Mendelian Randomization(MR) analysis. single-nucleotide polymorphisms used in our study were derived from genome-wide association studies (GWAS). The inverse variance weighted was the dominated method to evaluate the causality. The heterogeneity was validated by Cochran's Q test. The pleiotropy was validated by MR-Egger regression and MR-PRESSO. The stability and reliability of the results were illustrated by the ‘leave-one-out’sensitivity analysis. The MR results explored positive causal effects of BMI (OR: 1.021; 95% CI: from 1.016 to 1.027; *P* = 4.25 × 10^−15^) and WC (OR: 1.021; 95% CI: from 1.015 to 1.028; *P* = 1.65 × 10^−10^) on pancreatobiliary diseases. However, no causality existed between HC, WHR and pancreatobiliary diseases. This study reminded that general obesity and abdominal obesity required weight loss to prevent pancreatic biliary disease.

**Supplementary Information:**

The online version contains supplementary material available at 10.1007/s40519-023-01592-x.

## Introduction

Obesity, defined as a pathological condition that impairs health due to abnormal or excessive accumulation of lipids in adipose tissue, have been a global health crisis. According to a research, it is estimated that there are 603.7 million adults and 107.7 million children globally being obese [1]. Diagnosed at a body mass index (BMI) ≥ 30 kg/m^2^, obesity is regarded as a kind of chronic inflammation and is associated with the occurrence and development of many diseases [2]. A study indicated that BMI could increase risk of gallbladder and pancreatic cancer with a positive dose–response relationship [3]. In addition, another study found that non-biliary cancers were more associated with BMI than biliary cancers [4]. Previous studies maybe have reached a consensus that risk factors for pancreatic and biliary diseases included smoking, type 2 diabetes mellitus (T2DM), obesity and alcohol consumption and so on [5, 6]. However, it's worth noting that most of these literatures were based on observational data, which can only indicate the association and can not determine causal association between risk factors and outcomes due to the existence of confounding factors. Generally, the most common parameter to determine obesity is BMI. However, it might be inaccurate to assess obesity by BMI level alone because of the presence of abdominal obesity. It has been reported that it might be more effective to combine BMI with anthropometric characteristics such as waist circumference (WC), hip circumference (HC), and waist-to-hip ratio (WHR) when assessing obesity [7]. A few studies combined BMI and body type characteristics to assess obesity levels which were unable to establish a causal relationship between obesity and pancreatobiliary diseases.

Mendelian randomization (MR) analysis is an emerging epidemiological method employing genetic variation as an instrumental variables (IVs) [8, 9]. This method is similar to traditional randomized controlled trials(RCT), but can minimize the confounding factors of RCT [10], because the variables are distributed randomly and equally between the population, and that the genotypes are existence before the disease and independent of external factor after birth [11]. Therefore, it is widely used to explore causal relationships. In this study, single-nucleotide polymorphisms (SNPs) were employed as the IVs to conduct MR analysis to explore causal relationships between obesity (BMI, WC, HC, and WHR) and pancreatobiliary diseases.

## Materials and methods

### Study design

**Fig.1:**
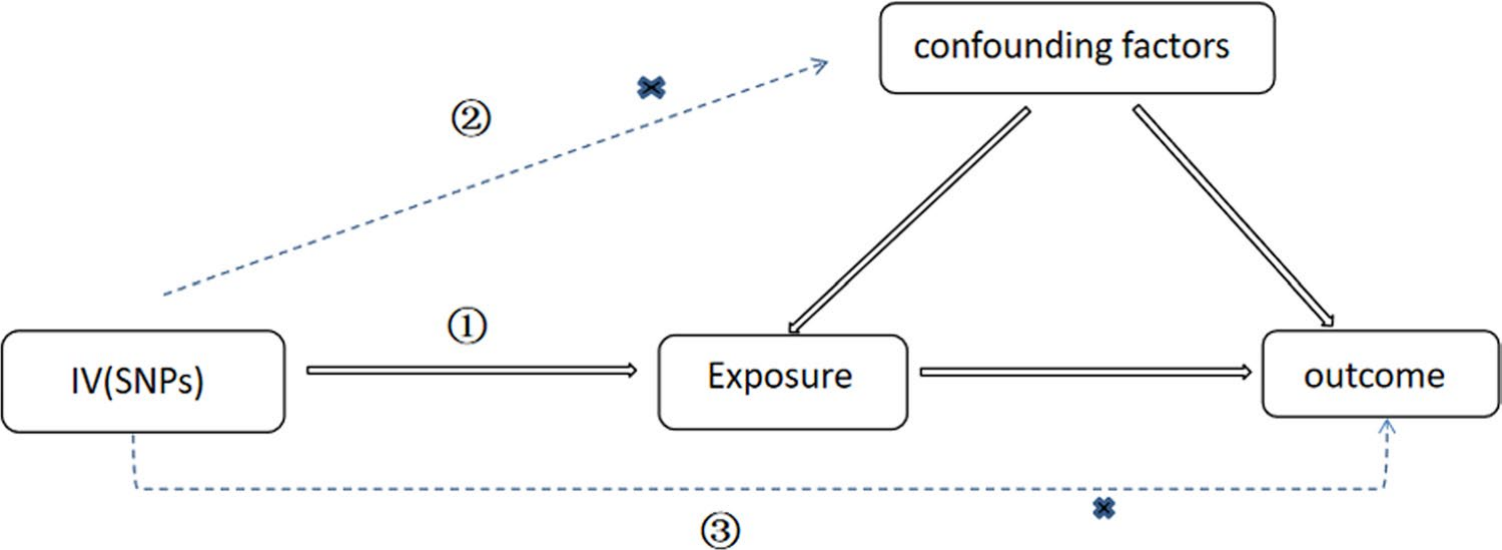
The schematic diagram of Mendelian Randomization

MR which can be carried out is based on the theory that because different genotypes determine different intermediate phenotypes which act as an individual's exposure, the association between the genotype and the disease can represent the effect of the exposure on the disease [12]. As shown in Fig. 1, SNPs worked as IVs to indirectly explore the relationship between exposure and outcome. However, in order to properly and reasonably deduce causal relationship between exposure and outcome, three important assumption must be met [13]:

1. The SNPs should be strongly associated with exposure.
2. The SNPS selected should have little to do with confounding factors.
3. The SNPs should influence results only through exposure but not the direct correlation.

**Fig.2:**
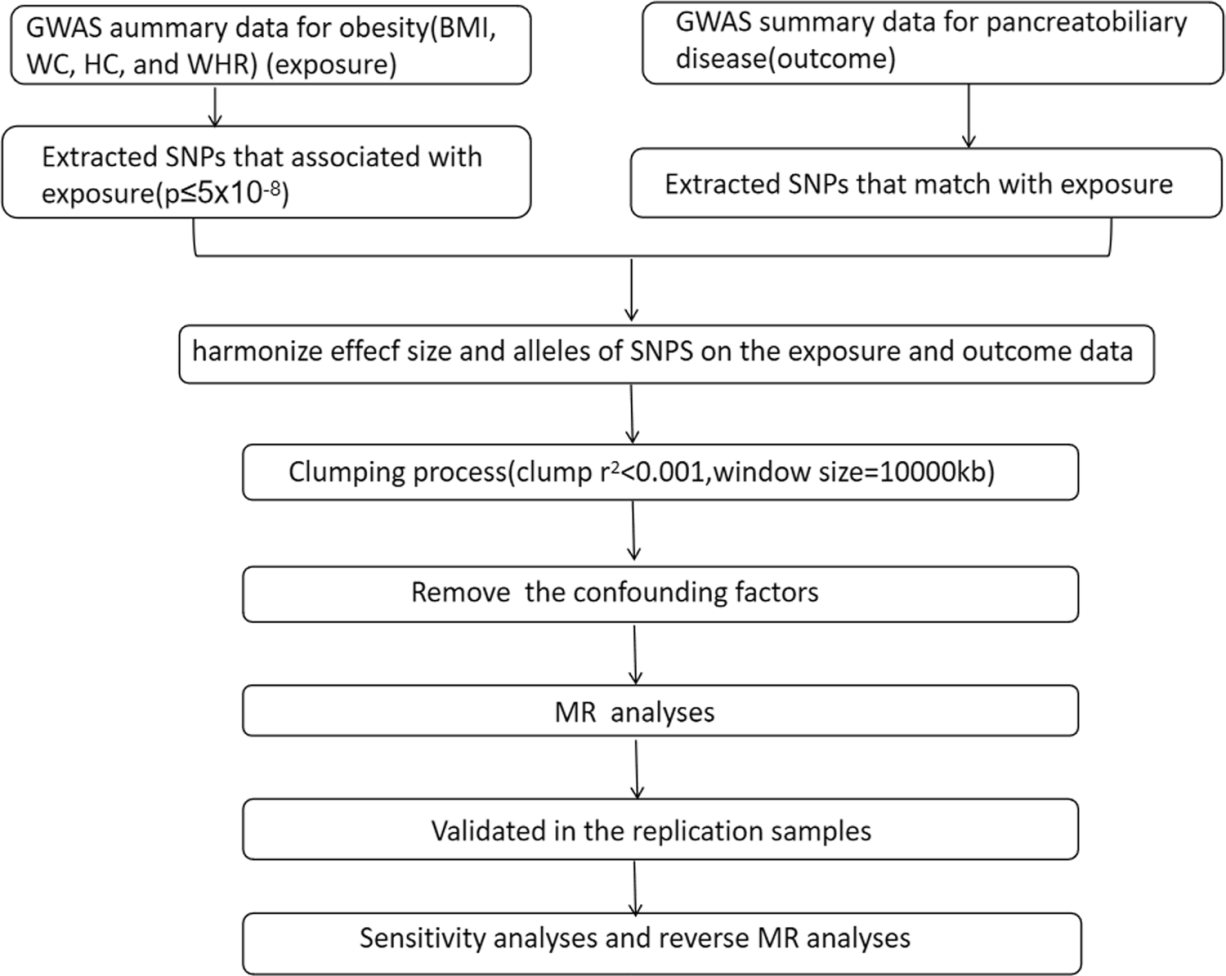
The flowchart of the study. *BMI* body mass index, *WC* waist circumference, *HC* hip circumference, *WHR* waist-to-hip ratio, *SNPs* ingle-nucleotide polymorphisms, *MR* Mendelian randomization

The flowchart of the study is shown in Fig. 2. The SNPs sassociated with BMI, WC, HC and WHR were employed to research the causality between obesity and pancreatobiliary diseases.

### Data sources

The data in our study were obtained from the genome-wide association studies (GWAS) datasets. Genetic variants for BMI were obtained from the GIANT Consortium based on GWAS with 339,224 participants and 2,555,511 SNPs [14]. The genetic variants for WC, HC and WHR, respectively, brought 232,101 (2,565,408 SNPs), 225,487 (2,542,663 SNPs) and 224,459 (2,562,516 SNPs) participants from GIANT Consortium into the study [15]. Genetic predictors of disorders of gallbladder, biliary tract and pancreas were obtained from UK Biobank, which enrolled 361,194 individuals (13,586,589 SNPs) and consisted of 13,922 cases and 347,272 controls. The ethical approval in our study was not required, because of available data in public GWAS data set (https://gwas.mrcieu.ac.uk).

### Instrumental variable

In our study, the following criteria were applied to select the IVs: (1) The SNPs associated with each genus at the locus-wide significance threshold (*P* ≤ 1.0 × 10^–8^) were extracted as potential IVs. the F-statistics were calculated with the following equation to assess the strength of each IV. To reduce the bias caused by weak IVs, weak IVs with *F* < 10 were excluded [16]. (2) The linkage disequilibrium (LD) was detected to guarantee that the SNPs were independent (*r*^2^ < 0.001,window size = 10,000 kb) and removed if containing LD. (3) The SNPs involved in the confounding factors were removed at Phenoscanner (http://www.phenoscanner.medschl.cam.ac.uk/).

$$F = \frac{{R^{2} * (N - 2)}}{{(1 - R^{2} )}}$$

$$R^{2} = \frac{{(2 \times \text{EAF} \times (1 - EAF) \times \text{beta}^{2} )}}{{\left[ {(2 \times \text{EAF} \times (1 - \text{EAF}) \times \text{beta}^{2} ) + (1 - \text{EAF}) \times N \times SE(\text{beta}^{2} )} \right]}}$$

where *R*^2^ the proportion of variance in the exposure explained by the genetic variants, *N* sample size, EAF the effect allele frequency, beta the estimated genetic effect on physical activity, SE (beta) the standard error of the genetic effect

### Statistical analysis

The inverse variance weighted (IVW), weighted median, MR-Egger, Simple mode and Weighted mode methods were applied to evaluate the causal associations between BMI, WC, HC, WHR, and pancreatobiliary diseases. IVW was the dominant analysis, which analyzed each Wald ratio and provided the most accurate estimate and was sensitivity to pleiotropy [17]. The heterogeneity was validated by Cochran's Q test. The MR-Egger regression methods and the MR-PRESSO were applied to conduct sensitivity analyses to examine whether the potential violation of the second and third assumptions of MR was existence[18]. MR-PRESSO global test and MR-PRESSO outlier test was used to examine the pleiotropy and remove the pleiotropy of IVs, respectively. There was no directional pleiotropy with the Egger-intercept of the linear regression close to 0 and horizontal pleiotropy of the IVs with P-value of MR-PRESSO global test > 0.05, then the exclusivity assumption could be considered to be valid[19]. The stability and reliability of the results were illustrated by the ‘leave-one-out’sensitivity analysis. All statistical analyses can be conducted by the ‘Two-Sample MR’package and MR-PRESSO packages in R (version 4.2.2) software. Statistically significant difference was considered at *P* < 0.05.

## Results

In our study, the characteristics of obesity were selected as risk factors for pancreatobiliary diseases for MR Analysis. Then, conversely, MR Analysis was performed with pancreaticobiliary disease as a risk factor and obesity characteristics as an outcome. According to the described selection criteria, there were 67 BMI-related SNPs with F ranging between 29.9 and 239.9, 34 WC-related SNPs with F ranging between 29.3 and 128.4, 66 HC-related SNPs with F ranging between 30.9 and 188.1, and 27 WHR-related SNPs with F ranging between 29.2 and 169.8 selected for MR analysis for pancreaticobiliary disease outcome. Details of the relevant SNPS for MR analysis were Presented in Tables S1–S4.

### Mendelian randomization analyses

**Fig. 3:**
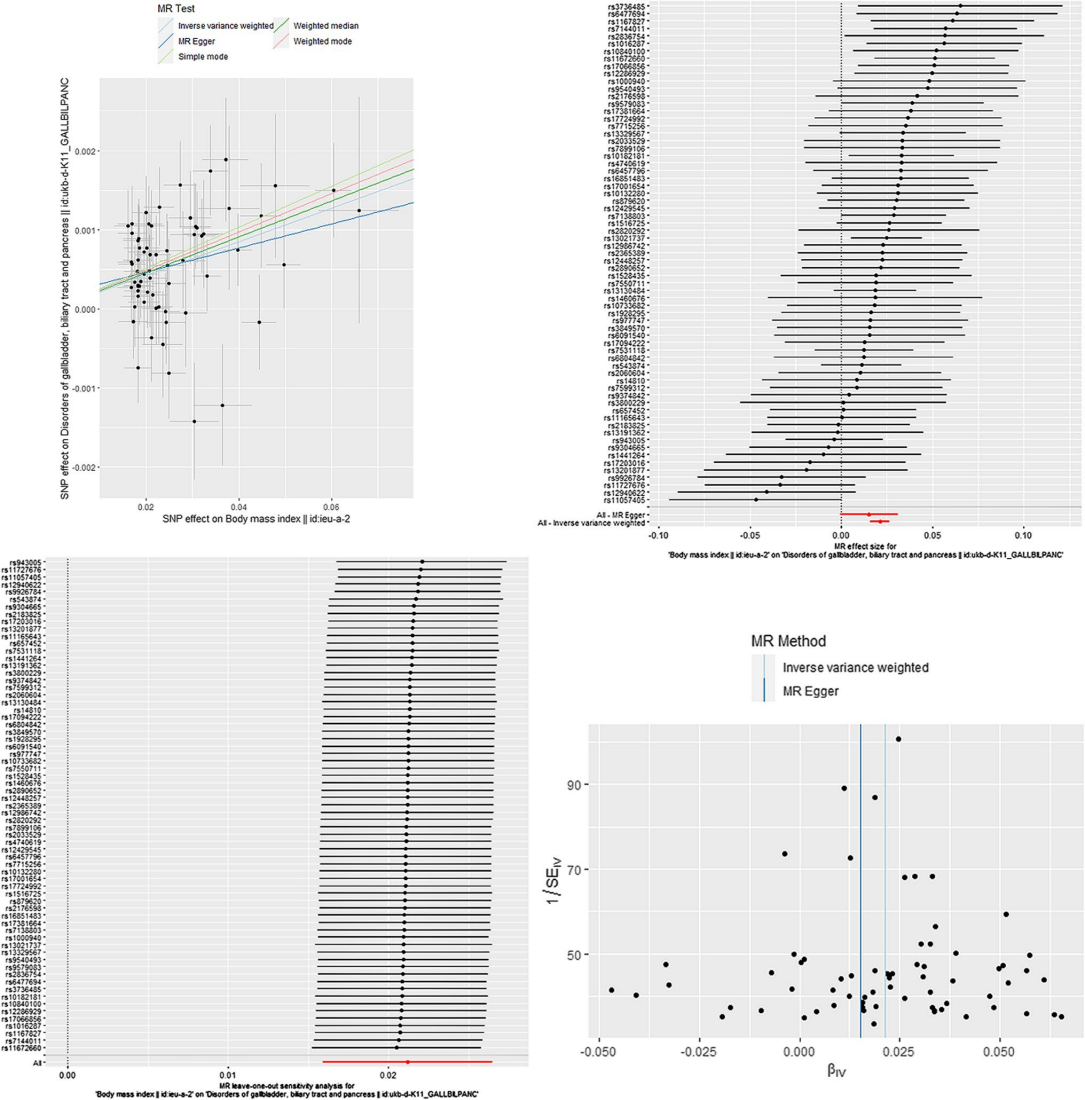
The MR results of BMI on pancreaticobiliary disease. Scatter plot about the causal effect of BMI on pancreaticobiliary disease. Forest plot for the overall causal effects of BMI on pancreaticobiliary disease. Leave-one-out analysis for the causal effect of BMI on pancreaticobiliary disease. Funnel plot of SNPs related to BMI and pancreaticobiliary disease

**Fig. 4:**
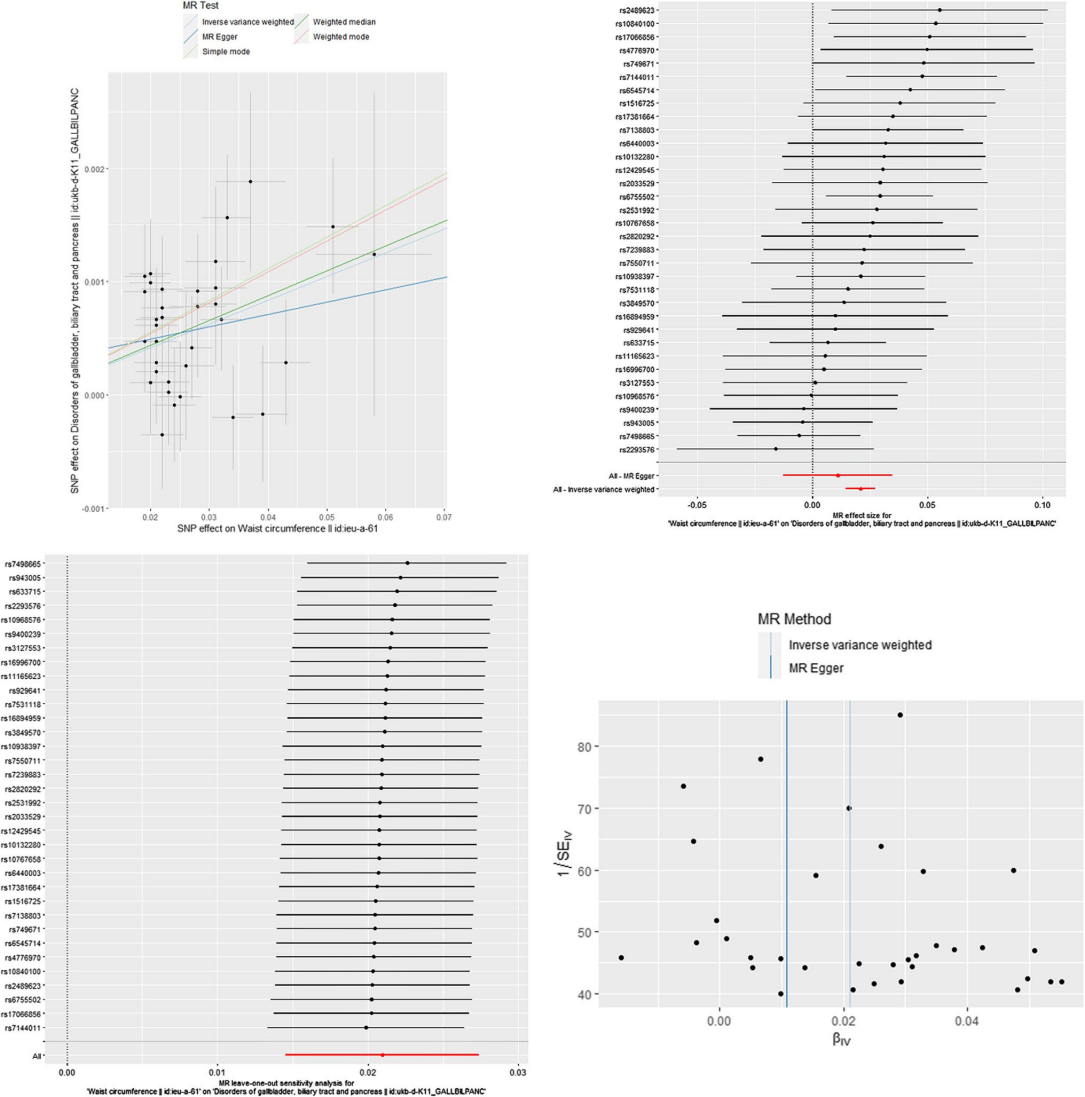
The MR results of WC on pancreaticobiliary disease. Scatter plot about the causal effect of WC on pancreaticobiliary disease. Forest plot for the overall causal effects of WC on pancreaticobiliary disease. Leave-one-out analysis for the causal effect of WC on pancreaticobiliary disease. Funnel plot of SNPs related to WC and pancreaticobiliary disease

Results presented in Table 1, which were reported as odds ratios (OR), made the casual relationship between obesity and pancreaticobiliary disease clear. As shown in Figs. 3 and 4, the IVW method indicated that BMI was causal risk factor of pancreaticobiliary disease (OR: 1.021; 95% CI: from 1.016 to 1.027; *P* = 4.25 × 10^−15^) with no heterogeneity (*Q* = 76.14, *P* = 0.1845) and WC was causal risk factor of pancreaticobiliary disease (OR: 1.021;95% CI: from 1.015 to 1.028; *P* = 1.65 × 10^−10^) with no heterogeneity (*Q* = 31.24, *P* = 0.5548). The results of HC, WHR and pancreaticobiliary diseases were listed in Table 1. However, the MR analysis indicated no causal relationship was existence between HC and WHC on pancreaticobiliary diseases as shown in the IVW result respectively (OR: 0.999; 95% CI: from 0.994 to 1.003; *P* = 0.585) and (OR: 1.009; 95% CI: from 1.001 to 1.018; *P* = 0.0206).

**Table1:** The Mendelian randomization analysis results with regard to causal effect of BMI, WC, HC, and WHR on pancreaticobiliary disease

Exposure	SNPs(n)	IVW			Weighted median		MR Egger				MR PRESSO	
		OR (95%CI)	Q (*P*-value)	*P*-value	OR (95%CI)	*P*-value	OR (95%CI)	Q (*P*-value)	Intercep (*P*-value)	*P*-value	No. of outlier	*Global Test* *P*-value
BMI	67	1.021 (1.016, 1.027)	76.1 (0.185)	4.25 × 10^–15^	1.023 (1.015, 1.030)	8.46 × 10^–9^	1.015 (0.999, 1.031)	75.4 (0.177)	0.00016 (0.431)	0.064	NA	0.2264
WC	34	1.021 (1.015,1.028)	31.2 (0.555)	1.65 × 10^–10^	1.022 (1.012, 1.032)	6.89 × 10^–6^	1.011 (0.9873, 1.035)	30.5 (0.543)	0.00028 (0.392)	0.3767	NA	0.5914
HC	66	0.999 (0.994,1.003)	78.5 (0.121)	0.643	0.995 (0.989,1.001)	0.121	1.009 (0.991,1.028)	77 (0.127)	− 0.0003 (0.266)	0.336	NA	0.119
WHR	27	1.009 (1.001,1.018)	29.3 (0.296)	0.0206	1.01 (0.999,1.022)	0.072	0.981 (0.947,1.015)	26.4 (0.387)	0.00073 (0.107)	0.285	NA	0.323

### Sensitivity analysis

The F-statistic for all IVs was >10, which meant that our estimate of the causal relationship between obesity and pancreaticobiliary disease was more accurate and precise. As shown in Table 1, no directional pleiotropy and horizontal pleiotropy was found for the analyze of BMI and WC. However, horizontal pleiotropy and heterogeneity were found by MR-PRESSO and Cochran's *Q* test when it came to HC and WHR with *P*_MR-PRESSO_ = 0.0194, *Q* = 92.7, *P*_Q test_ = 0.0204 and *P*_MR- PRESSO_ = 0.019, *Q* = 48.2, *P*_Q test_ = 0.0139, respectively. MR-PRESSO outlier test was carried out. Actually, several potentially pleiotropic SNPs were excluded. Specifically speaking, rs1053593 and rs4239437 were excluded from the analysis of HC; rs12549058, rs17451107 and rs2287019 were excluded from the analysis of WHR. As shown in Table 1, Similar results were observed with no pleiotropy and heterogeneity when these SNPs were excluded from the analysis. Scatter-plot revealed there was no causal relationship between WHR and pancreaticobiliary disease, although IVW indicated OR: 1.009, 95% CI: from 1.001 to 1.018, *P* = 0.0206.

## Discussion

The purpose of our study was to investigate the relationship between obesity and pancreaticobiliary disease by two-sample Mendelian randomization analysis. Although BMI played an important role in assessing obesity, it was difficult to distinguish between abdominal and peripheral fat [20]. Therefore, BMI and body type characteristics (WC, HC, and WHR) was applied to assess the causal effect of obesity on pancreaticobiliary disease in our study, which made it clear that BMI and WC, rather than HC and WHR, had causal effects on pancreaticobiliary disease.

Pancreatic diseases are a large category of diseases occurring in the pancreas. Patients with pancreatic disease and obesity are common in clinical practice. Previous studies have shown that WC was associated with the development of acute pancreatitis [21, 22], meaning that for every 10 cm increase in waist circumference, the probability of acute pancreatitis increased by 40% [22]. Navina et al. found that the increased risk of acute pancreatitis due to increased abdominal circumference may be related to increased retroperitoneal, peripancreatic, or intrapancreatic volume [23]. There are a few literatures on chronic pancreatitis and obesity. A study focused on impact of pediatric acute recurrent and chronic pancreatitis showed that obese or overweight children were diagnosed with chronic pancreatitis at an older age, which meant obesity might be a long-term proinflammatory state that eventually progressed to a chronic inflammatory state [24]. Obesity is regarded as a kind of chronic inflammation and can increase the levels of TNFα, IL-1β, IL-6, and IL-18 within adipose tissue and systemically, such as through inflammasome activation in macrophages [25]. An animal study showed that obesity caused deficiency of PGC-1α in the pancreas, which significantly enhanced NF-kB-mediated up-regulation of IL-6 in pancreas, resulting in serious inflammatory responses [26].

Biliary tract disease is a general term of gallbladder cancer, ampulla cancer, cholelithiasis, cholecystitis and so on and may be related with body fatness through oxidative stress, increased inflammation and so on [4]. A meta-analysis of BMI, WC, WHR, and gallbladder disease risk showed a summary relative risk of 1.46 for every 10 cm increase in WC and 1.44 for every 0.1 unit increase in WHR. Even within the "normal" BMI range, the risk almost twofold increase [27]. According to a European cohort, obesity, especially abdominal obesity, was associated with the risk of gallbladder cancer [28]. Interesting, obesity was thought to be associated with cholangiocarcinoma in Western countries, but had no effect on its development in Asia population [29].

Although obesity was shown in our study to be associated with pancreatobiliary diseases. However, there were still some limitations. Firstly, this study was primarily based on European population, whether similar results would be obtained in other population was unknown. Secondly, because of the different etiopathogenesis and heterogeneous of the pancreatobiliary diseases, and unclear criteria which had been specifically taken into account from the genome-wide association studies (GWAS) datasets obtained from the GIANT Consortium and from UK Biobank, it was hard to conduct subgroup analysis in pancreatobiliary diseases, making our results seemed to be excessive. Nevertheless, our results give a new insight, especially regarding which types of obesity requires weight loss to prevent pancreatic biliary disease”.

## Conclusion

This MR study revealed that body shape traits were associated with pancreatobiliary diseases and health benefits of losing weight within reasonable target to prevent pancreatic biliary disease still remained attractive.

## Supplementary Information

Below is the link to the electronic supplementary material.

Supplementary file1 (XLSX 29 KB)

## Data Availability

Data supporting the findings of this study are available within the paper and its supplementary information files.
